# The association between boredom and mind-wandering weakens with increasing depressive symptom severity

**DOI:** 10.1186/s40359-026-05362-z

**Published:** 2026-08-14

**Authors:** Johannes P.-H. Seiler, Florian Müller-Dahlhaus, Flávio Kapczinski, Tiago Figueiredo

**Affiliations:** 1 Institute of Physiology, Focus Program Translational Neurosciences, University Medical Center of the Johannes Gutenberg University Mainz, Duesbergweg 6, 55128 Mainz, Germany; 2 Department of Psychiatry and Psychotherapy, University Medical Center of the Johannes Gutenberg University Mainz, Untere Zahlbacher Straße 8, 55131 Mainz, Germany; 3 Department of Psychiatry, Universidade Federal do Rio Grande do Sul, Porto Alegre, Rio Grande do Sul Brazil; 4 Instituto Cognus de Ensino e Pesquisa (ICEP), SGAS 915, 69/70. Asa Sul, Federal District, Brasília, Brazil

**Keywords:** Boredom, Depression, Mind-wandering, Spontaneous thought

## Abstract

**Background:**

Boredom is an aversive state commonly elicited by monotonous or insufficiently engaging situations. Mind-wandering has been proposed as one possible response to boredom, but the association between these phenomena may vary with depressive symptom severity, particularly when mind-wandering is habitual, excessive, or poorly regulated. We therefore examined whether depressive symptom severity moderates the association between state boredom and habitual excessive mind-wandering in psychiatric patients.

**Methods:**

This cross-sectional study examined self-reported state boredom, depressive symptoms, and habitual excessive mind-wandering in 94 psychiatric outpatients spanning multiple diagnostic categories and age groups.

**Results:**

Depressive symptom severity was reliably associated with state boredom across diagnostic categories (R_P_=0.623, *p* < 0.001). In addition, when studying the moderating effects of depressive symptom severity on the association between boredom and excessive mind-wandering, we observed a positive association of boredom and mind-wandering that progressively weakened with increasing degrees of depression (interaction of depression and boredom: β=−0.184, *p* = 0.039, ΔR^2^ = 0.033).

**Conclusions:**

Our findings indicate a robust correlation of state boredom and depression in various mental health disorders. Moreover, higher severity of individual depressive symptoms is associated with an attenuation of the coupling between state boredom and excessive mind-wandering tendencies. Replication in larger and more homogeneous samples is required to determine the generalizability of these observations.

**Trial registration:**

Not applicable.

**Supplementary Information:**

The online version contains supplementary material available at 10.1186/s40359-026-05362-z.

## Introduction

Boredom is an aversive mental state characterized by cognitive disengagement and difficulty sustaining satisfying attention [1–4]. It is commonly elicited by monotonous situations and conditions of low information content [5–8]. Due to its aversive experience, boredom is thought to act as a cognitive signal that drives exploration of alternative and novel sources of information [9, 10]. While boredom under conditions of mental health is often followed by immediate actions to alleviate this experience [9, 11, 12], various psychiatric disorders have been linked with elevated and prolonged levels of boredom experience [13–15]. In this context, *state boredom* describes the transient experience of boredom in a given, temporally limited situation, whereas *trait boredom* describes the temporally stable tendency of a person to experience boredom across different situations [1, 12, 16].

Although both trait and state boredom have been associated with psychiatric disorders [14, 16–18], trait boredom has been criticized for conceptual heterogeneity [19] and inconsistent construct validity [20–22]. In contrast, state boredom provides a more temporally specific measure [11, 22], and has recently been linked to clinical outcomes [23] and sensory processing [24]. We therefore focus on state boredom throughout this study.

Responses to state boredom include seeking stimulation externally in one’s environment [2, 8, 9, 25], but may also include mechanisms to explore internal stimuli [7] through engagement in interoception [26] and mind-wandering [27–30]. Mind-wandering is commonly defined as a shift of attention away from the ongoing task towards self-generated, task-unrelated, often stimulus-independent thought [29]. Mind-wandering is reliably elicited under conditions that reduce external cognitive demand – such as monotony, low sensory information content, and waning motivation – and it is modulated by affective and control-related factors such as fatigue and differences in mood [31]. Theoretical accounts therefore describe mind-wandering as one possible response to boredom when access to more rewarding external activities is constrained [7, 30, 32].

Mind-wandering can support adaptive functions such as future planning and internal exploration, but excessive or poorly regulated mind-wandering has also been associated with psychopathology, including depression and anxiety [27, 33]. For instance, habitual excessive mind-wandering in depression is often unintentional and constrained to stereotypical and repetitive content [27]. Relatedly, rumination – a hallmark symptom of depressive disorder [34, 35] – also describes an inflexible type of ideation with repetitive and negative thought content. In this study, we focus on excessive forms of spontaneous thought rather than adaptive or creative ideation, using specific questionnaires to assess excessive forms of mind-wandering and study its link to boredom [36].

Despite the individual linkages between mind-wandering, boredom and depression, the mechanisms that determine if mind-wandering alleviates or perpetuates boredom remain insufficiently specified [4, 9, 37, 38]. In particular, it is unclear how different psychometric features, such as differences in depressive symptoms, influence the relationship between state boredom and excessive mind-wandering. Two opposing scenarios appear plausible: On the one hand, depressive symptoms may strengthen the association between boredom and excessive mind-wandering, because both tend to increase with negative affect. On the other hand, depressive symptoms may constrain spontaneous thought towards repetitive and ruminative content, thereby weakening its association with boredom.

Here, we tested these scenarios by investigating the relationship between state boredom, depressive symptoms and mind-wandering in a broad transdiagnostic sample of psychiatric outpatients, spanning adolescence and adulthood. Our primary hypothesis was that depressive symptom severity would moderate the association between state boredom and habitual excessive mind-wandering, such that the association would be weaker at higher levels of depressive symptoms. 

Based on this hypothesis, we asked three questions: (i) How homogeneous is the profile of boredom experience in different psychiatric disorders? (ii) How strongly are state boredom and its subdimensions associated with differences in depressive symptom severity? (iii) Does depressive symptom severity moderate the cross-sectional association between state boredom and habitual excessive mind-wandering? To address these questions, we used standardized self-report measures to quantify state boredom, depressive symptoms, and habitual excessive mind-wandering in individual patients, and used a continuous moderation model to evaluate the interaction between boredom and mind-wandering under varying depressive symptom severity.

## Materials and methods

### Participants

For this cross-sectional study, we recruited a broad sample of 94 psychiatric outpatients who consulted a private mental health institute in the Federal District of Brazil (Instituto Cognus de Ensino e Pesquisa, Brazil) for diagnostic clarification and therapeutic advice. The sample comprised 55 men (58.5%) and 39 women (41.5%) with a mean age of 21.9 years (SD = 11.9 years). Age and sex were assessed through self-reports. Participants were recruited for the study irrespective of diagnosis, leading to a broad cohort spanning various mental disorders, children and adolescents as well as adults (49, i.e. 52.1% patients with < 18 years, and 45, i.e. 47.9% patients with ≥ 18 years). Specifically, our sample comprised 85 patients with a single psychiatric diagnosis (*unipolar depression/major depressive disorder*: *n* = 8, *bipolar disorder*: *n* = 6, *attention deficit hyperactivity disorder* (ADHD): *n* = 47, *autism spectrum disorder* (ASD): *n* = 16, *other* comprising anxiety disorders, psychotic disorders and obsessive-compulsive disorders: *n* = 8), and 9 patients diagnosed with multiple psychiatric disorders (*co-morbid*). Diagnoses were established by qualified psychiatrists according to the Diagnostic and Statistical Manual of Mental Disorders, Fifth Edition (DSM-5). Demographic information by diagnostic group is provided in Supplementary Table 1. Medication status and time since first psychiatric diagnosis were not assessed for this study.

Participants were eligible if they were fluent in Portuguese and provided the required consent or assent. Exclusion criteria were active neurological or genetic disorders and severe intellectual impairment defined as an estimated IQ < 70 (verified through neuropsychological testing independent from the study).

We included all eligible patients admitted during the recruitment period; consequently, the diagnostic composition of the sample was not balanced by design. The sample size was based on the feasibility of recruitment and on a previous exploratory clinical study from our group [23], rather than on an a priori power calculation.

The study was approved by the Ethics Committee of the Federal University of Rio de Janeiro (CAAE: 74921623.3.0000.5263). All procedures were conducted in accordance with the Declaration of Helsinki. All adult participants provided written informed consent. For participants younger than 18 years, a parent or legal guardian provided written informed consent. Participation was discussed jointly with the child or adolescent and the parent or legal guardian, and participation took place only when both agreed.

### Data acquisition

After admission and consent, participants completed various self-report scales assessing individual state boredom (Multidimensional State Boredom Scale, MSBS), affective symptom severity (either Children’s Depression Inventory (CDI) for participants younger than 18 years, or Depression, Anxiety and Stress Scale (DASS) for adults), and habitual excessive mind-wandering (Mind Excessively Wandering Scale, MEWS). Additional assessments were administered for clinical diagnostic purposes but were not included in the present analyses.

### Psychometric assessments

All questionnaires employed in this study are well-established instruments with previously demonstrated reliability and validity. To further characterize the psychometric properties of the questionnaires most central to the present study, internal consistency (Cronbach’s alpha) was additionally estimated for the MSBS and MEWS using item-level responses from the current sample. Because the DASS-21 and CDI have already been extensively validated in Brazilian Portuguese populations, we report their published psychometric characteristics rather than re-estimating internal consistency.

#### Multidimensional State Boredom Scale (MSBS)

The Multidimensional State Boredom Scale [11] (MSBS) contains 29 items to assess situational boredom experience. State boredom is assessed in sum (total score) and on five subdimensions: Disengagement, High Arousal, Low Arousal, Inattention, and Time Perception. Items are rated from 1 ("strongly disagree") to 7 ("strongly agree"), with higher total scores indicating higher levels of boredom. The MSBS has been successfully adapted and validated in several languages, showing broad internal and external validity, as indicated by reliable associations with sensation-seeking [22, 39–41]. We used the Brazilian Portuguese version [40]. In the original Brazilian validation study, the MSBS showed good internal consistency (Cronbach’s alpha: total = 0.901; disengagement = 0.680; high arousal = 0.709; low arousal = 0.850; inattention = 0.859; time perception = 0.832). In the present clinical sample, the MSBS total score showed high internal consistency (Cronbach’s α = 0.909), whereas subscale estimates ranged from lower to good internal consistency (Cronbach’s alpha, disengagement = 0.844; high arousal = 0.682; low arousal = 0.839; inattention = 0.820; time perception = 0.756).

#### Depression, Anxiety and Stress Scale (DASS-21)

The 21-item version of the Depression, Anxiety and Stress Scale [42, 43] (DASS) was administered to adults (≥ 18 years). Items are rated from 0 ("did not apply to me at all") to 3 ("applied to me very much or most of the time"). We used the Brazilian Portuguese version, for which the validation studies reported high internal consistency (Cronbach’s alpha: depression: 0.92, anxiety: 0.86, stress: 0.90) [44, 45]. In the present study, 45 adult patients completed the CDI.

#### Children’s Depression Inventory (CDI)

The Children’s Depression Inventory [46] (CDI) was developed to assess depressive symptoms in school-aged children and adolescents and was derived from Beck’s Depression Inventory [47]. We used a Brazilian version of the CDI [48, 49] in participants younger than 18 years. Brazilian validation studies [48] have reported CDI internal consistency estimates ranging from Cronbach’s alpha = 0.73 to 0.91. In the present study, 49 child and adolescent patients completed the CDI.

#### Mind-Wandering Scale (MEWS)

Habitual excessive mind-wandering was assessed with the Brazilian Portuguese version of the Mind Excessively Wandering Scale [36, 50] (MEWS). The MEWS is a retrospective self-report measure of habitual mind-wandering in which higher scores indicate a higher tendency to engage extensively in spontaneous thought processes that distract individuals from their current environment. The original questionnaire was developed in adults with ADHD [36], and subsequent cross-cultural adaptations demonstrated good psychometric properties [50] and high internal consistency (Cronbach’s alpha ranging from 0.93 to 0.97). In the present clinical sample, the MEWS also demonstrated excellent internal consistency (Cronbach’s alpha = 0.943).

### Statistical analysis

All analyses were conducted in MATLAB^®^ using the Statistics and Machine Learning Toolbox (The MathWorks Inc., Natick, Massachusetts, USA; version R2022a). All data were pseudonymized before analyzing. We first conducted exploratory diagnostic-group and age-group comparisons of state boredom. We then pooled participants across diagnostic categories to examine dimensional associations among state boredom, depressive symptoms, and habitual excessive mind-wandering in a transdiagnostic manner.

#### Data processing and handling of missing data

Questionnaire total and subscale scores were calculated according to the relevant scoring procedures (see above). Participants who omitted one or more items required for a score were excluded only from analyses involving that score. Therefore, the effective sample size varied across analyses. Diagnostic and demographic comparisons of state boredom included all 94 participants. Correlations between MSBS scores and depressive symptom severity included 89 participants with complete MSBS and depression data. The primary moderation model included 89 participants with complete MSBS, MEWS, and depression data. The exploratory quartile analysis (see Methods below for details) used 88 of these 89 participants, with one participant omitted solely to create four equally sized groups of 22.

#### Pooling the CDI and DASS data to obtain a standardized depression score

Because the CDI and DASS-Depression scale were administered to mutually exclusive age groups, we standardized each score within its respective age group before pooling. Specifically, CDI scores were converted to z-scores within participants younger than 18 years, and DASS-Depression scores were converted to z-scores within adults. This approach assumed that the CDI and DASS-Depression scale assess a shared construct of depression. A sufficient degree of convergent validity between both questionnaires to justify this procedure is supported by two observations: First, robust empirical evidence shows that both scales correlate with independent measures of depression [44, 46, 49], supporting indirect convergence through a shared validated anchor construct [51]. Second, a strong correlation between CDI and DASS-Depression scores within given clinical samples completing both questionnaires further support conceptual overlap between the two scales [52, 53]. The pooled score represented a relative metric of depressive symptom severity within each age group rather than providing an absolute depression metric across developmental stages. Accordingly, the pooled score was used as a dimensional index in our analyses, and instrument-specific analyses with CDI and DASS only were conducted to support our pooled analyses.

#### Correlation analyses

We examined associations among boredom, affective symptoms, and excessive mind-wandering using Pearson correlations, and corroborated these analyses with Spearman rank correlations. We report effect estimates, sample sizes, and raw p-values. Statistical significance was evaluated at a two-sided alpha level of 0.05, with the multiplicity procedures described below.

#### Correction for multiple testing

For correlation analyses, we used Bonferroni-adjusted thresholds or Holm-Bonferroni corrections, as specified in the respective figure legends and the Results section. The continuous moderation model was treated as the principal inferential model in the revised manuscript and was not adjusted for the exploratory correlations or subgroup visualizations. The moderation analysis was hypothesis-driven but not preregistered and should therefore be considered exploratory.

#### Continuous moderation model

We used multiple linear regression to model MEWS total scores as a function of mean-centered MSBS total scores, mean-centered pooled standardized depression scores, and their interaction. In this moderation model, the interaction coefficient quantified to which extent the association between state boredom and habitual excessive mind-wandering varied with depressive symptom severity. Assumptions of linear regression were verified by inspection of residual normality (Q-Q plot, Lilliefors test: *p* = 0.073), homoscedasticity (residuals vs. fitted), and absence of multicollinearity (variance inflation factor VIF = 1.647), confirming the suitability of the linear model. To estimate the significance of the impact of the regressors on mind-wandering, we reported standardized weights, confidence intervals, p-values and t-statistics for each regressor. To further estimate the contribution of the different regressors on mind-wandering, we computed the contributions of each model parameter, quantified as the absolute values of the products of the respective regression weight and the individual parameter values. In addition, we computed semi-partial R^2^, total model R^2^, and the change in R^2^ relative to an additive model without the interaction.

#### Sensitivity analyses

To evaluate the robustness of the primary moderation model, two sets of sensitivity analyses were performed. First, the moderation analysis was repeated after additionally adjusting for age, sex, and ADHD diagnosis (ADHD vs. non-ADHD) as covariates. Second, because depressive symptom severity was assessed using different questionnaires in participants younger than 18 years (CDI) and adults (DASS-Depression), the moderation analysis was repeated separately within the CDI and DASS subsamples.

#### Sensitivity power analysis

To facilitate interpretation of the observed moderation effect, a sensitivity power analysis based on the non-central F distribution was performed. Assuming a significance level of α = 0.05 and 80% statistical power, the minimum detectable interaction effect was estimated and expressed as Cohen’s f^2^ together with the corresponding increase in explained variance (ΔR²).

#### Correlation of boredom and mind-wandering in groups with varying depressive symptom severity

To further assess and visualize the relationship between boredom and mind-wandering in patients with varying extent of depressive symptoms, we conducted an exploratory analysis, grouping 88 of the 89 patients providing full questionnaire data, into four equally sized groups of 22 participants according to ranked standardized depressive symptom severity (standardized depression score boundaries: z-score [−1.563; −0.928; −0.083; 0.763; 2.243]). One participant was omitted solely to obtain equal group sizes. This subgroup analysis was descriptive and was not used for the primary statistical inference. The continuous moderation model included all 89 complete cases (see above).

#### Conditional simple-slope estimates

Following the continuous interaction model, we estimated the conditional association between MSBS and MEWS at the mean and at one standard deviation below and above the mean of the pooled depression index. Predictors were mean-centered before the interaction term was calculated. Conditional slopes, 95% confidence intervals, t-statistics, and p-values were obtained from the fitted coefficient covariance matrix.

#### Statistical tests

Age-group differences were examined using two-sided Wilcoxon rank-sum tests, and diagnostic-group differences were examined using Kruskal-Wallis tests. These comparisons were exploratory because the diagnostic groups were comparably small and unequal and because multiple MSBS outcomes were examined.

## Results

### Exploratory comparison of state boredom across diagnostic groups

We recruited a transdiagnostic sample of psychiatric outpatients (see Methods, Supplementary Table 1, Supplementary Fig. 1 A) and assessed affective symptoms, state boredom and habitual excessive mind-wandering using a battery of self-report assessments (see Methods).

Self-reported affective symptoms and mind-wandering tendencies quantified as MEWS scores varied across diagnostic groups, with the highest levels in patients diagnosed with major depressive disorder or bipolar disorder, as expected (Supplementary Fig. 1B-F).

**Fig. 1:**
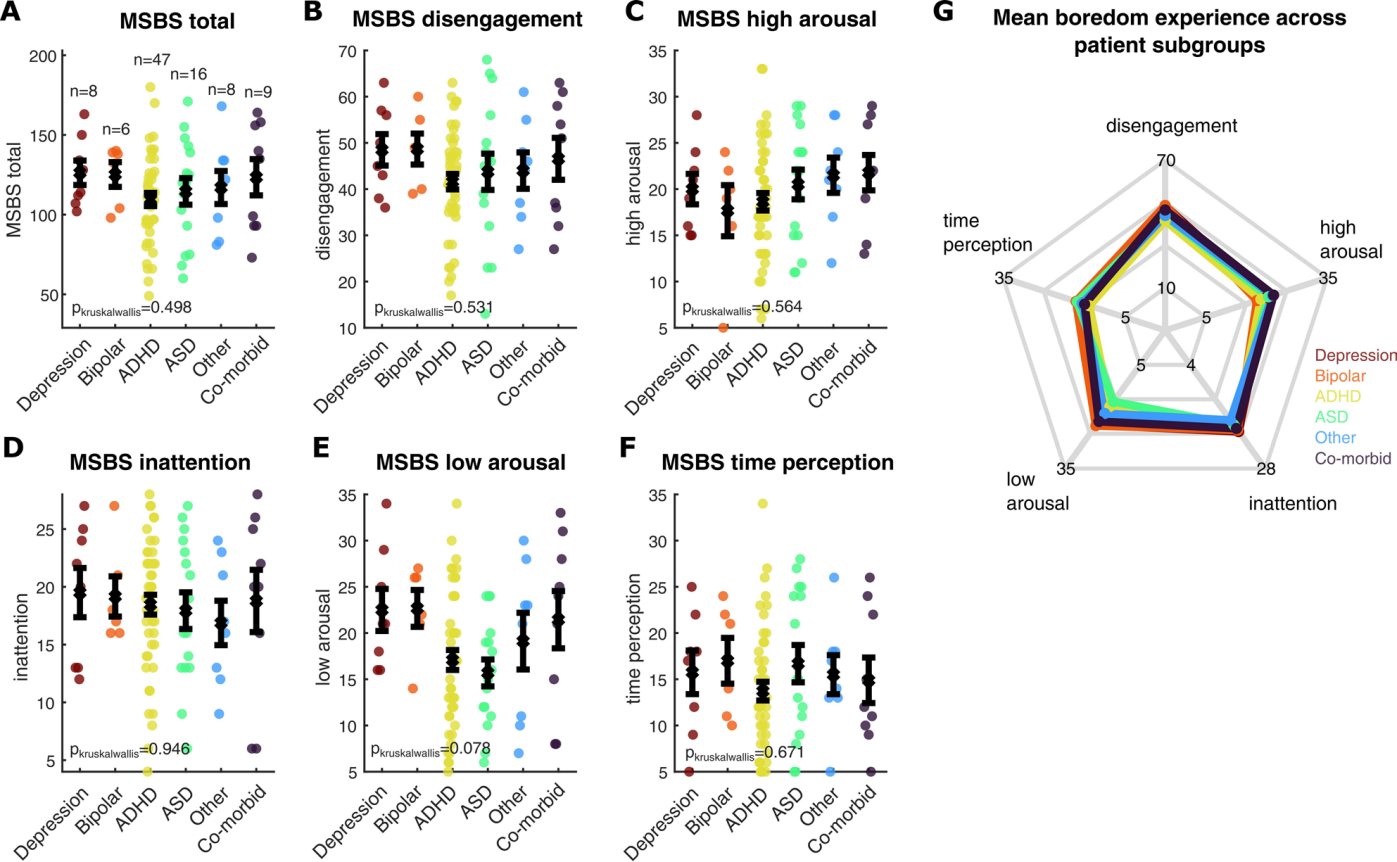
Exploratory comparison of state boredom across diagnostic groups: **A** Distribution of total state boredom scores (MSBS total, Multidimensional State Boredom Scale) across all diagnosis groups in our transdiagnostic patient sample (*n* = 94 patients in total; for diagnostic and demographic details see Supplementary Table 1). Reported p-values refer to Kruskal-Wallis tests across groups. Reported n-values describe the number of patients per diagnostic group. **B-F** Equivalent analysis for all sub-scales of the MSBS. **G** Spider plot showing the mean state boredom profile in all sub-dimensions of the MSBS across diagnostic groups

We did not detect a statistically significant difference in state boredom quantified as MSBS total scores across diagnostic groups (Fig. 1A; *n* = 94 patients, Kruskal-Wallis test across groups, *p* = 0.498). Likewise, no statistically detectable differences between diagnosis groups were observed for the boredom subdimensions of disengagement, high arousal, inattention, or time perception (Fig. 1B-F; *n* = 94 patients, Kruskal-Wallis test across groups: Disengagement *p* = 0.531, High Arousal *p* = 0.564, Inattention *p* = 0.946, Time Perception *p* = 0.671). The differences in the low arousal subscale also did not reach the significance threshold (*p* = 0.078), although the observed scores varied descriptively across groups.

Thus, the average state boredom profiles appeared comparable across diagnostic groups in the present sample (Fig. 1G), with no statistically significant group differences for most boredom dimensions and only a trend towards differences in low arousal.

### Exploratory comparison of state boredom between adults and adolescents

We next conducted an exploratory comparison of MSBS scores between participants younger than 18 years (*n* = 49) and adults (*n* = 45).

**Fig. 2:**
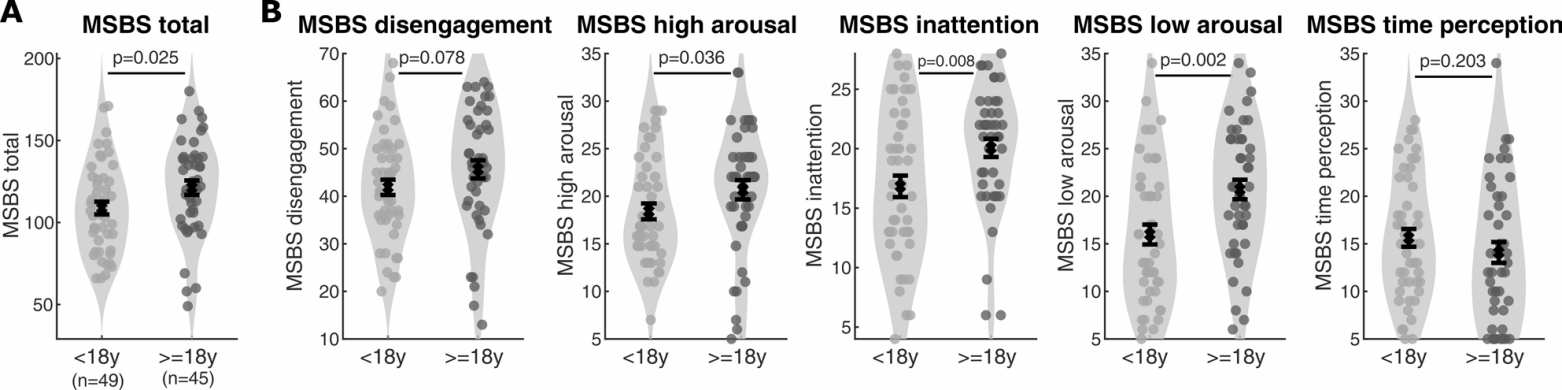
Exploratory comparison of state boredom across age groups: **A** Total MSBS scores in adult patients (age ≥ 18 years, *n* = 45) and children or adolescent patients (age < 18 years, *n* = 49), pooled across all diagnostic groups (MSBS: Multidimensional State Boredom Scale). Reported p-value refers to a Wilcoxon rank sum. **B** Equivalent exploratory analyses for the five MSBS subscales. No correction for multiple testing was applied.

We observed moderately higher levels of overall state boredom in adult patients (Fig. 2A; Wilcoxon rank sum test: MSBS total *p* = 0.025). In subscale comparisons, higher boredom scores in adults were also observed for high arousal, inattention, and low arousal, whereas disengagement and time perception did not differ significantly (Fig. 2B; Wilcoxon rank sum test: MSBS Disengagement *p* = 0.078, High Arousal *p* = 0.036, Inattention *p* = 0.008, Low Arousal *p* = 0.002, Time Perception *p* = 0.203). These observations suggest a moderate increase in boredom experience in psychiatric patients with higher age.

### State boredom is reliably associated with depressive symptoms

Next, we examined the association between state boredom and depressive symptom severity by calculating the correlations between MSBS scores and depression questionnaire scores.

**Fig. 3:**
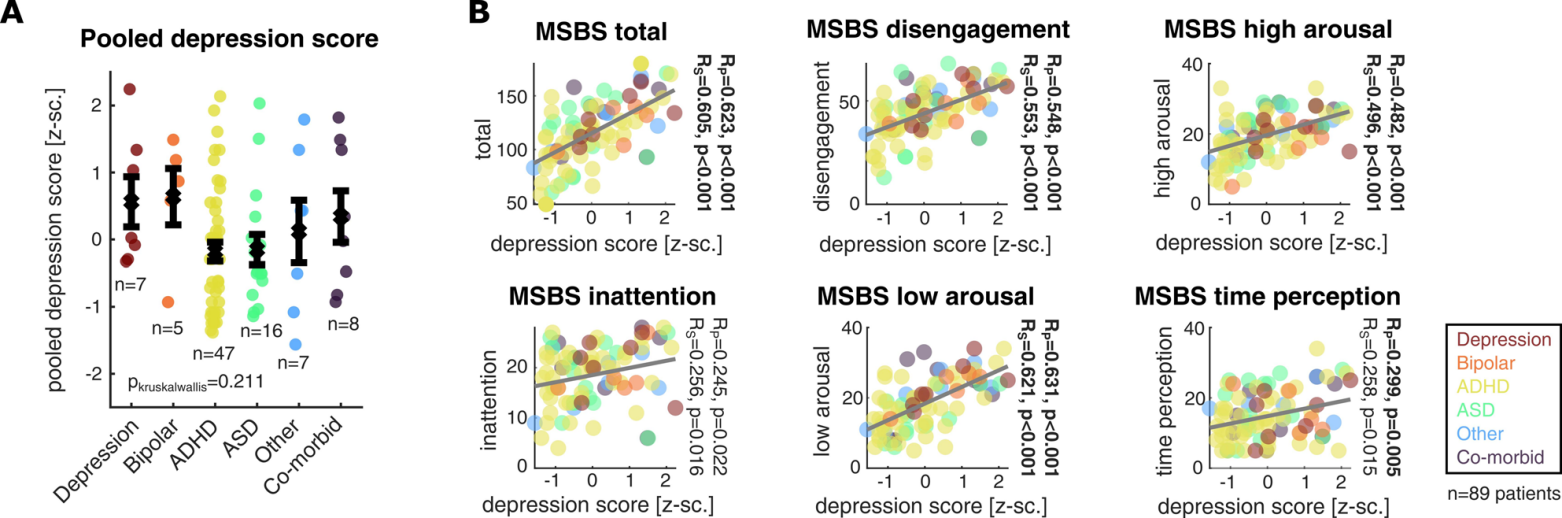
Depressive symptom severity is associated with elevated state boredom: **A** CDI scores in participants younger than 18 years and DASS-Depression scores in adults were standardized within their respective age groups and pooled to create a relative depression index (see Methods). This standardized depression score showed broad distribution across the different diagnosis groups with highest levels in patients diagnosed with major depressive disorder or bipolar disorder. **B** Pearson and Spearman correlations between the pooled depression scores and the MSBS total and subscale scores (*n* = 89 patients, R_P_: Pearson correlation coefficient, R_S_: Spearman correlation coefficient). Individual data points are colored by diagnostic group. Bold values denote associations meeting the Bonferroni-adjusted significance threshold (*p* < 0.008)

Specifically, we pooled the data of all patients and computed a standardized depression score based on z-normalization within each age-group, integrating the CDI and DASS-Depression scores (see Methods for details). This score provided a relative rather than an absolute measure of depressive symptom severity for all patients in our sample, setting the individual symptom severity in relation to the average severity of similarly aged individuals. As expected, this standardized depression score tended to be highest for patients with affective disorders (major depressive disorder and bipolar disorder; Fig. 3A; *n* = 90 participants with depression ratings, Kruskal-Wallis test between diagnosis groups: *p* = 0.211).

We then correlated the pooled standardized depression scores with MSBS scores observing a substantial positive association (Fig. 3B; *n* = 89 patients with standardized depression scores and MSBS total, R_P_=0.623, *p* < 0.001, R_S_=0.605, *p* < 0.001). The correlation to depressive symptom severity was particularly pronounced for the sub-dimensions of disengagement, high arousal and low arousal (Disengagement: R_P_=0.548, *p* < 0.001, R_S_=0.553, *p* < 0.001, High Arousal: R_P_=0.482, *p* < 0.001, R_S_=0.496, *p* < 0.001, Inattention: R_P_=0.245, *p* = 0.022, R_S_=0.256, *p* = 0.016, Low Arousal: R_P_=0.631, *p* < 0.001, R_S_=0.621, *p* < 0.001, Time Perception: R_P_=0.299, *p* = 0.005, R_S_=0.258, *p* = 0.015). Color-coding the data of single patients based on their psychiatric diagnosis and testing their correlation independently, supported that this positive association of state boredom and depression was prevalent for all patients in our sample, spanning different disorders (Supplementary Fig. 2).

The positive association between MSBS total scores and depressive symptoms was also observed when the instruments were analyzed separately (Supplementary Fig. 3; MSBS total vs. CDI: *n* = 49 patients, R_P_=0.642, *p* < 0.001; MSBS total vs. DASS-Depression: *n* = 45 patients, R_P_=0.622, *p* < 0.001).

In adults, MSBS total scores were also associated with DASS-Anxiety but not with DASS-Stress (see Supplementary Fig. 3, last two columns of correlation matrix; MSBS total vs. DASS-Anxiety: *n* = 45 patients, R_P_=0.516, *p* = 0.017; MSBS total vs. DASS-Stress: *n* = 45 patients, R_P_=0.352, *p* = 0.446).

Together, these correlation analyses support a reliable and consistent association between depressive symptom severity and boredom experience in our transdiagnostic sample.

### Depressive symptom severity moderates the association between boredom and mind-wandering

We next sought to test whether depressive symptom severity moderates the association between state boredom and habitual excessive mind-wandering.

**Fig. 4:**
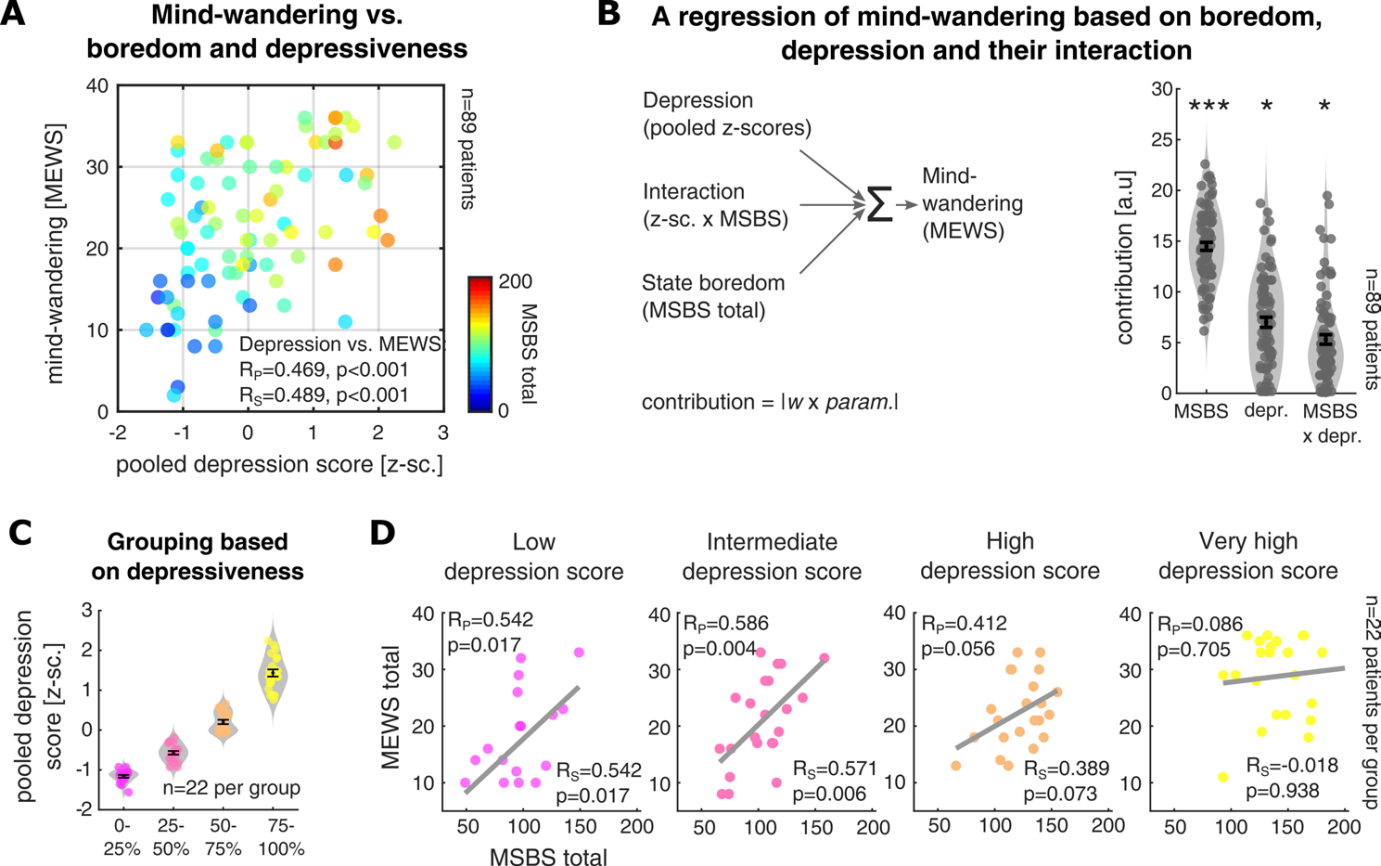
A weakened association between boredom and mind-wandering with increasing depressive symptom severity: **A** Association between the pooled standardized depression scores and MEWS scores (*n* = 89 patients, R_P_: Pearson correlation coefficient, R_S_: Spearman correlation coefficient). Datapoints are color-coded by the MSBS total scores. **B** Primary linear regression, modeling individual MEWS scores with MSBS total scores, pooled standardized depression scores, and their interaction. Parameter contributions and significance levels are shown for descriptive purposes (see Methods; *: *p* < 0.05, ***: *p* < 0.001); full coefficients and confidence intervals are reported in the text. **C** To visualize the moderating effect of depressive symptom severity on the association between boredom and excessive mind-wandering, we grouped our cohort into four equally sized groups based on their pooled standardized depression scores (see Methods). **D** For each group (low, intermediate, high, and very high depressive symptom severity), we independently computed the correlation between MSBS total scores and MEWS scores, observing positive correlations in the groups with low and intermediate depression, but an increasing decorrelation with higher and very high levels of depressive symptoms (*n* = 22 patients per group, R_P_: Pearson correlation coefficient, R_S_: Spearman correlation coefficient)

To this end, we first correlated the pooled standardized depression scores of all patients to the total MEWS scores (Fig. 4A), and observed a significant positive correlation (*n* = 89 patients, R_P_=0.469, *p* < 0.001, R_S_=0.489, *p* < 0.001). Based on the previously observed consistency of correlations between depressive symptoms and the various subdimensions of state boredom, we used the MSBS total scores as a measure of overall boredom, finding that boredom was positively associated with more excessive mind-wandering, as expected (see color gradient in Fig. 4A). We then tested in how far differences in age might account for the variance in excessive mind-wandering, depression severity and boredom. We found only a moderate positive correlation between age and MEWS scores (*n* = 89 patients, R_P_=0.304, *p* = 0.005), whereas depression and boredom were not significantly correlated with age in our sample (*n* = 89 patients, MSBS vs. age: R_P_=0.140, *p* = 0.180, standardized depression score vs. age: R_P_=−0.060, *p* = 0.575).

To directly investigate in how far depressive symptom severity moderates the association between state boredom and extensive mind-wandering, we conducted a multiple linear regression, modeling individual MEWS scores as a linear combination of MSBS total scores, pooled standardized depression scores, and the interaction, i.e. the product of both metrics (Fig. 4B; Methods). We compared the absolute contribution of these three regressors on mind-wandering (see Methods), and observed a significant effect of all three (*n* = 89 patients, full model R^2^ = 0.380; regression coefficients: MSBS: standardized β = 0.424, 95%-CI=[0.201 0.648], *p* < 0.001, t(85) = 3.801, semi-partial R^2^ = 0.109, standardized depression score: standardized β = 0.254, 95%-CI=[0.025 0.483], *p* = 0.013, t(85) = 2.529, semi-partial R^2^ = 0.037, MSBS x depression: standardized β=−0.184, 95%-CI=[−0.358 −0.010], *p* = 0.039, t(85)=−2.102, semi-partial R^2^ = 0.034). Adding the interaction between boredom and depressive symptoms modestly but significantly improved model fit relative to an alternative additive model (F-change(1,85) = 4.42, *p* = 0.039), increasing the explained variance from R^2^ = 0.347 to R^2^ = 0.380 (ΔR^2^ = 0.033), representing 3.3% of total outcome variance and approximately 8.7% of the variance explained by the full model. A sensitivity power analysis further indicated that with *n* = 89 and α = 0.05, the primary moderation model had 80% power to detect an incremental interaction effect of approximately Cohen’s f²=0.090 or larger. The observed interaction was slightly smaller (f²=0.053; ΔR²=0.033), indicating limited power for an effect of the observed magnitude. These analyses indicate that despite a modest effect magnitude, depressive symptom severity significantly moderates the association between state boredom and excessive mind-wandering.

To test the robustness of this moderation, we conducted a sensitivity analysis with an extended regression model adjusting for age, sex, and ADHD status. In the adjusted model, the interaction between MSBS and pooled standardized depression scores remained negative and statistically significant (see Supplementary Table 2; standardized β = −0.197, 95%-CI=[− 0.361 − 0.033], *p* = 0.019). The direction and magnitude were similar to those in the primary unadjusted model. In additional instrument-specific sensitivity analyses, considering the CDI and DASS scores independently, the interaction between MSBS scores and depressive symptom severity also remained negative (Supplementary Table 2; CDI subgroup: standardized β = −0.278, 95%-CI=[− 0.510 − 0.047], *p* = 0.020; DASS subgroup: standardized β = −0.103, 95%-CI=[− 0.379 0.172], *p* = 0.452). Together, these analyses show consistent directionality of the interaction between state boredom and depressive symptom severity across control models, supporting the robustness of the observed moderation effect.

To explore the moderation effect in more depth and visualize it, we grouped our sample into four evenly sized groups based on their pooled standardized depression scores and computed the correlation between MSBS total scores and MEWS scores independently for each group (Fig. 4C, Methods, *n* = 22 patients per group). We observed that groups with low depressive symptom severity showed a comparably strong correlation between MSBS and MEWS scores, whereas this positive correlation gradually declined with increasing depressive symptom severity (Fig. 4D; *n* = 22 patients per group, Low depression: R_P_=0.542, *p* = 0.017, R_S_=0.542, *p* = 0.017, Intermediate depression: R_P_=0.586, *p* = 0.004, R_S_=0.571, *p* = 0.006, High depression: R_P_=0.412, *p* = 0.056, R_S_=0.389, *p* = 0.073). In the group of patients reporting the highest levels of depressive symptoms, we did not detect a statistically significant correlation (Very high depression: *n* = 22 patients, R_P_=0.086, *p* = 0.705, R_S_=−0.018, *p* = 0.938).

To corroborate this exploratory subgroup-based analysis, we conducted additional continuous moderation analysis with the data of our full sample, probing the simple slopes between MSBS scores and MEWS scores at different levels of standardized depression scores, in order to estimate the relationship between boredom and excessive mind-wandering over varying levels of depressive symptom severity (see Methods). In accordance with our subgroup analysis, we observed a significant association of MSBS and MEWS scores at low and medium depressive symptom severity, whereas we did not detect a statistically significant association of boredom and mind-wandering at high depressive symptom severity (mean-1SD stand. depression score (−0.992): slope = 0.179, 95%-CI=[0.098 0.259], *p* < 0.001, t(85) = 4.427; mean stand. depression score (0.016): slope = 0.125, 95%-CI=[0.059 0.190], *p* < 0.001, t(85) = 3.772; mean+1SD stand. depression score (1.024): slope = 0.071, 95%-CI=[−0.016 0.157], *p* = 0.107, t(85) = 1.629).

Together, these findings indicate that the association between state boredom and excessive mind-wandering becomes progressively weaker with increasing depressive symptom severity.

## Discussion

In this cross-sectional study, we used self-report assessments in a broad cohort of psychiatric outpatients to investigate the interplay between state boredom, depressive symptoms and excessive mind-wandering. We did not detect clear diagnosis-specific differences in state boredom within this exploratory sample. Moreover, depressive symptom severity was associated with both boredom and excessive mind-wandering. Exploring the link between boredom, depressive symptoms and mind-wandering in more detail, we observed a weakening of the association between boredom and excessive mind-wandering in patients with higher depressive symptoms. Although the observed moderation effect was based on cross-sectional self-report data and does not establish causal mechanisms or distinguish between alternative explanations, we discuss plausible candidate cognitive mechanisms and the implications of these findings below.

### Depressive symptoms and boredom

While previous studies have largely focused on investigating the link between psychopathology and boredom on a temporally stable trait-level [15, 16, 18, 54], increasing evidence suggests that also temporally dynamic state boredom has a strong association with a person’s mental health status, allowing for instance to predict the length of psychiatric treatment duration [23]. In this study, we expanded this aspect by investigating the link between depression and state boredom, finding that the extent of state boredom is associated with the severity of depressive symptoms. Interestingly, this association was not only observed in patients with affective disorders but was shared across the full transdiagnostic sample.

The overall levels of state boredom in our patient sample were comparably high, with MSBS total scores being approximately twice as high as in non-clinical control participants [23], and we did not detect statistically significant differences across diagnostic groups. Depression scores across patient groups in our sample were also considerable, corresponding to a moderate severity of Major Depressive Disorder [42, 43, 55], and habitual mind-wandering was elevated to approximately five-fold of the MEWS scores observed in non-clinical controls [36]. These findings suggest that depressive symptoms, as a general factor of many psychiatric disorders [56–58], represent a transdiagnostic dimension closely associated with heightened experience of boredom and mind-wandering tendencies. Several non-specific processes may contribute to this association. For instance, elevated boredom in individuals with higher depressive symptoms may partly reflect broader psychopathological processes such as anhedonia, fatigue, or generalized negative affect rather than boredom-specific mechanisms [59]. Future studies combining behavioral and experience-sampling approaches may help disentangle these overlapping constructs.

### Mind-wandering in depression

Different psychiatric conditions may involve different responses to boredom [60]. In this respect, externalizing mental disorders, such as ADHD, may primarily react to boredom with sensation-seeking and externally directed search for stimulation [15, 24, 25], whereas internalizing mental disorders, such as depression, may primarily react to boredom with internally directed processes including excessive mind-wandering [30, 61]. Consistent with this idea and other research documenting an intricate relationship between spontaneous thought processes and depressive symptomatology [33, 62], we found that depressive symptoms are positively correlated to the extent of mind-wandering in our patient sample.

Mind-wandering by nature is heterogeneous: It may be deliberate or unintentional, adaptive or maladaptive, and positive or negative in content. Depression is typically characterized by more frequent ideation and by rigid, negative thought content [27, 35]. In particular, depressive patients often report rumination, defined as involuntary repetitive and passive thoughts about symptoms of distress [63, 64]. Thus, mind-wandering in depression is assumed to be constricted by strong automatic constraints that impair the adaptiveness and diversity of generated ideas and may thus contribute to higher levels of boredom. Experience-sampling studies further suggest a bidirectional relationship between mood and mind-wandering: While sadness tends to precede mind-wandering episodes, negative content of mind-wandering subsequently predicts states of reduced mood [65]. This bidirectional association suggests that mind-wandering may not only serve as a marker of depressive cognition but may also actively contribute to the maintenance of depressive symptoms through a self-reinforcing cycle of negative spontaneous thought.

### Depression severity moderates the link between boredom and mind-wandering

Although depressive symptoms were overall positively associated with both boredom and excessive mind-wandering, our moderation analysis indicated that the association between boredom and excessive mind-wandering became progressively weaker with increasing depressive symptom severity, suggesting that depressive symptoms may alter the coupling between spontaneous thought and boredom.

This moderation may be interpreted in light of theoretical links between mind-wandering and creativity [66]. Flexible mind-wandering has been proposed to facilitate the generation of novel associations that support creative thought and foster adaptive responses to monotonous situations [27, 67–69]. Accordingly, creativity has been reported to be negatively associated with boredom [24, 70], suggesting that flexible spontaneous thought may contribute to alleviating boredom. Consequently, inflexibel and constrained thought patterns [71–73] with stereotypical negative content [71, 74, 75] are characteristic of depression and may shift mind-wandering towards non-creative thought trajectories that do not generate sufficient stimulation, thus perpetuating boredom and weakening the association between internal thought processes and boredom. Because creativity was not assessed in the present study, this interpretation remains speculative and should be examined directly in future research.

## Possible physiological underpinnings

Another speculative interpretation of the linkage between boredom, mind-wandering and depressive symptoms concerns altered interoceptive processing. Recent work linked boredom to alterations in interoceptive processing [26] involving the insular cortex [76–78], a region also implicated in depression [79–81]. Altered, less reliable interoceptive processing may contribute to both affective dysregulation and impaired engagement with internal and external stimuli, hence linking depressive symptoms, boredom, and spontaneous thought. A speculative possibility is that while physiological interoception may stimulate productive mind-wandering by providing the brain with meaningful sensory information from within the body, pathological aberrations in interoception, characteristic for depression, may lead to misinterpreted or noisy bodily signals that constrain spontaneous thought. Under such conditions, mind-wandering may become dominated by perseverative negative content that is less efficient in alleviating boredom. Future neurophysiological studies combining assessments of interoception and experience sampling will be required to test these proposed mechanisms.

### Limitations

Several limitations should be considered when interpreting the present findings. Boredom, depressive symptoms, and related psychometric measures were assessed using standardized self-report questionnaires, making the findings susceptible to common-method variance as well as reporting biases common in clinical contexts [82]. Moreover, the study design was cross-sectional and did not include behavioral, or physiological measures, restricting the possible analyses of the data. Medication status and treatment history were not systematically considered and may also have influenced individual symptom profiles.

The study sample was comparably small, diagnostically heterogeneous, and strongly weighted towards ADHD, limiting the generalizability of our findings and potentially obscuring disorder-specific effects. As psychiatric disorders likely differ in the expression of depressive symptoms, associations between specific depressive symptom dimensions, boredom, or mind-wandering may have remained undetected. Accordingly, diagnosis-specific comparisons should be considered exploratory. Likewise, age-related differences should be interpreted cautiously, as age group, diagnostic composition, and the use of different depression measures were partly confounded and therefore cannot be fully disentangled.

While our sensitivity analyses supported robustness of the main moderation effect, the observed interaction between boredom and depressive symptom severity was smaller than the minimum effect detectable with 80% power in the available sample, and should therefore be interpreted cautiously until replicated in additional studies with larger and more balanced patient samples.

Pooling age-adjusted depression scores across the CDI and DASS assumed sufficient construct equivalence across developmental stages and provided a relative rather than absolute measure of depressive symptom severity. Furthermore, partial conceptual overlap between depressive symptoms and specific boredom dimensions, particularly low arousal and inattention, may have contributed to the observed associations, which may therefore partly reflect broader affective and attentional processes.

The MEWS assesses a habitual tendency towards excessive mind-wandering rather than the content, intentionality, or adaptiveness of spontaneous thought, whereas the MSBS assesses current state boredom. Consequently, our findings cannot determine whether mind-wandering occurred concurrently with, or in response to, boredom, nor which characteristics of spontaneous thought underlie the observed moderation effect.

Finally, the cross-sectional study design does not permit causal inference. Experimental and longitudinal studies with additional neurocognitive metrics will be required to determine the mechanisms underlying the observed associations.

## Conclusion

Taken together, this exploratory cross-sectional study provides evidence for a close interplay of depressive symptoms, boredom and spontaneous thought processes in psychiatric patients. Higher depressive symptom severity was associated with higher state boredom and more pronounced habitual mind-wandering. In addition, increasing depressive symptom severity was coupled to a a weakening of the association between mind-wandering and boredom. These observations set a starting point for further studies to replicate these effects and investigate the interplay of boredom, affect and spontaneous thought processes on a mechanistic level.

## Supplementary Information

Supplementary Material 1.

## Data Availability

The data of this study and the code to analyze it are available from the corresponding authors upon reasonable request.
