# Supplementary material for: The association between boredom and mind-wandering weakens with increasing depressive symptom severity
Source: BMC Psychol. 2026 Aug 14;14:1191. doi: 10.1186/s40359-026-05362-z (PMC13474920; doi:10.1186/s40359-026-05362-z)
Supplement: Supplementary file 1 [file 40359_2026_5362_MOESM1_ESM.pdf]

The association between boredom and mind-wandering weakens with increasing depressive symptom severity

Johannes P.-H. Seiler[1], Florian Müller-Dahlhaus[2], Flávio Kapczinski[3], Tiago Figueiredo[4]

[1] Institute of Physiology, Focus Program Translational Neurosciences, University Medical Center of the Johannes Gutenberg University Mainz, Duesbergweg 6, 55128 Mainz, Germany

[2] Department of Psychiatry and Psychotherapy, University Medical Center of the Johannes Gutenberg University Mainz, Untere Zahlbacher Straße 8, 55131 Mainz, Germany

[3] Department of Psychiatry, Universidade Federal do Rio Grande do Sul, Porto Alegre, Rio Grande do Sul, Brazil

[4] Instituto Cognus de Ensino e Pesquisa (ICEP), Federal District, Brazil

Correspondence concerning this article should be addressed to Tiago Figueiredo, SGAS 915, 69/70. Asa Sul. Zip Code: 70390-150, Brasília-DF, Brazil, or to Johannes P.-H. Seiler, Institute for Physiology, Focus Program Translational Neurosciences, University Medical Center of the Johannes Gutenberg University, Mainz, Germany. E-mail: tiagofigueiredosf@gmail.com, johseile@uni-mainz.de

- Supplementary information -

Supplementary Figures

Supplementary Figure 1

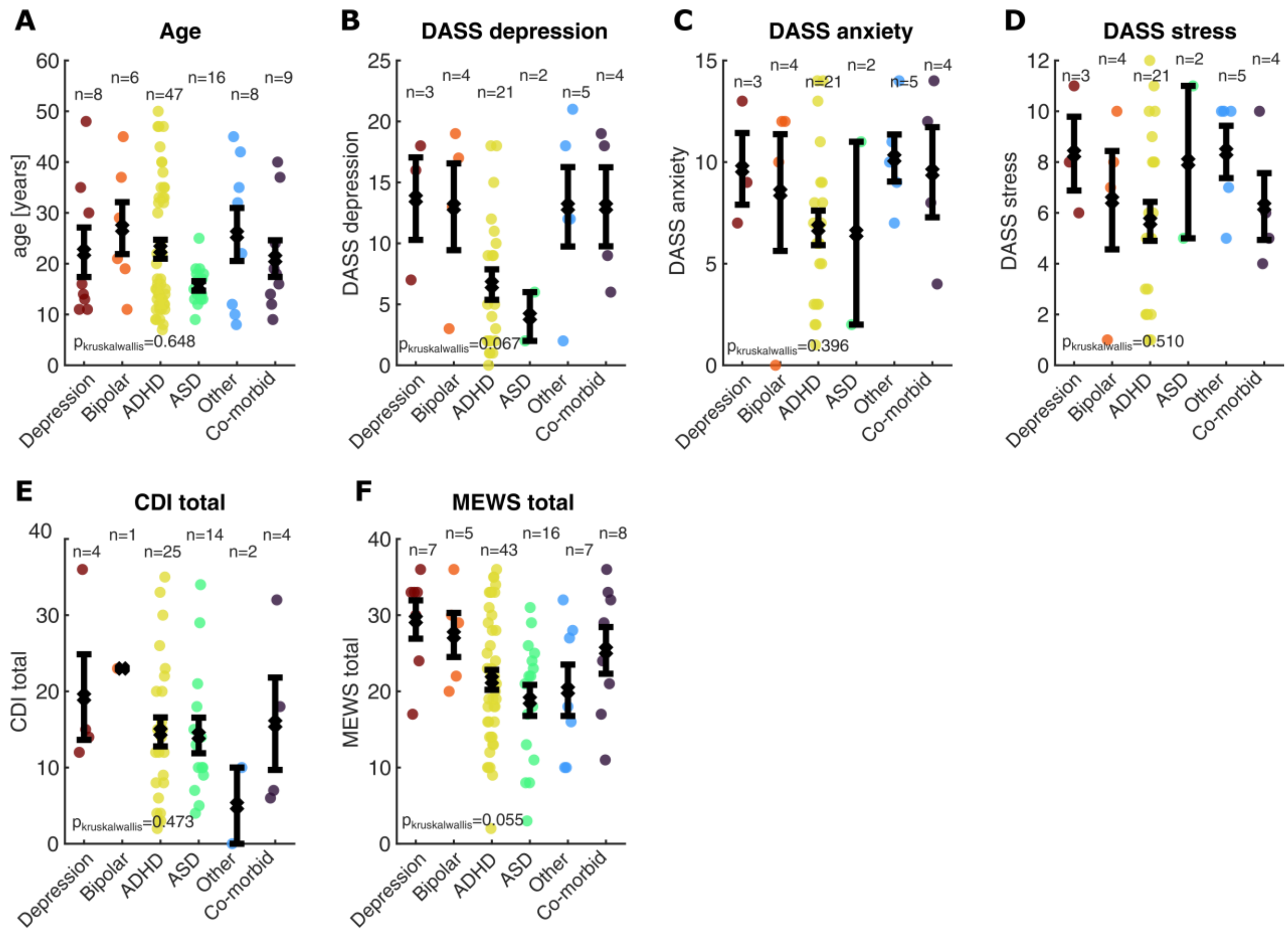

Supplementary Figure 1 – Distributions of age and psychometric reports across psychiatric diagnosis groups: (**A**) Age distributions across diagnostic groups in the transdiagnostic patient sample. The sample covered children, adolescents and adults in typical age ranges. The reported p-value refers to a Kruskal-Wallis test across diagnostic groups. Reported n-values indicate the number of patients per group. (**B**) Distributions of DASS depression scores across adult patients. Because the DASS was administered only to adults, the subgroup sample sizes differ from the complete diagnostic sample (see Methods). ADHD and ASD patients show a trend of lower depressive symptoms. (**C,D**) Equivalent analysis for the anxiety and stress subscales of the DASS, showing no statistically significant differences across groups. (**E**) CDI ratings among children and adolescents (<18 years, see Methods). (**F**) MEWS scores show a trend of elevation in unipolar depression and bipolar disorder, while ADHD and ASD tend to show lower levels of mind-wandering. Abbreviations: ADHD: Attention Deficit Hyperactivity Disorder; ASD: Autism Spectrum Disorder; DASS: Depression, Anxiety and Stress Scale; CDI: Children's Depression Inventory; MEWS: Mind Excessively Wandering Scale.

Supplementary Figure 2

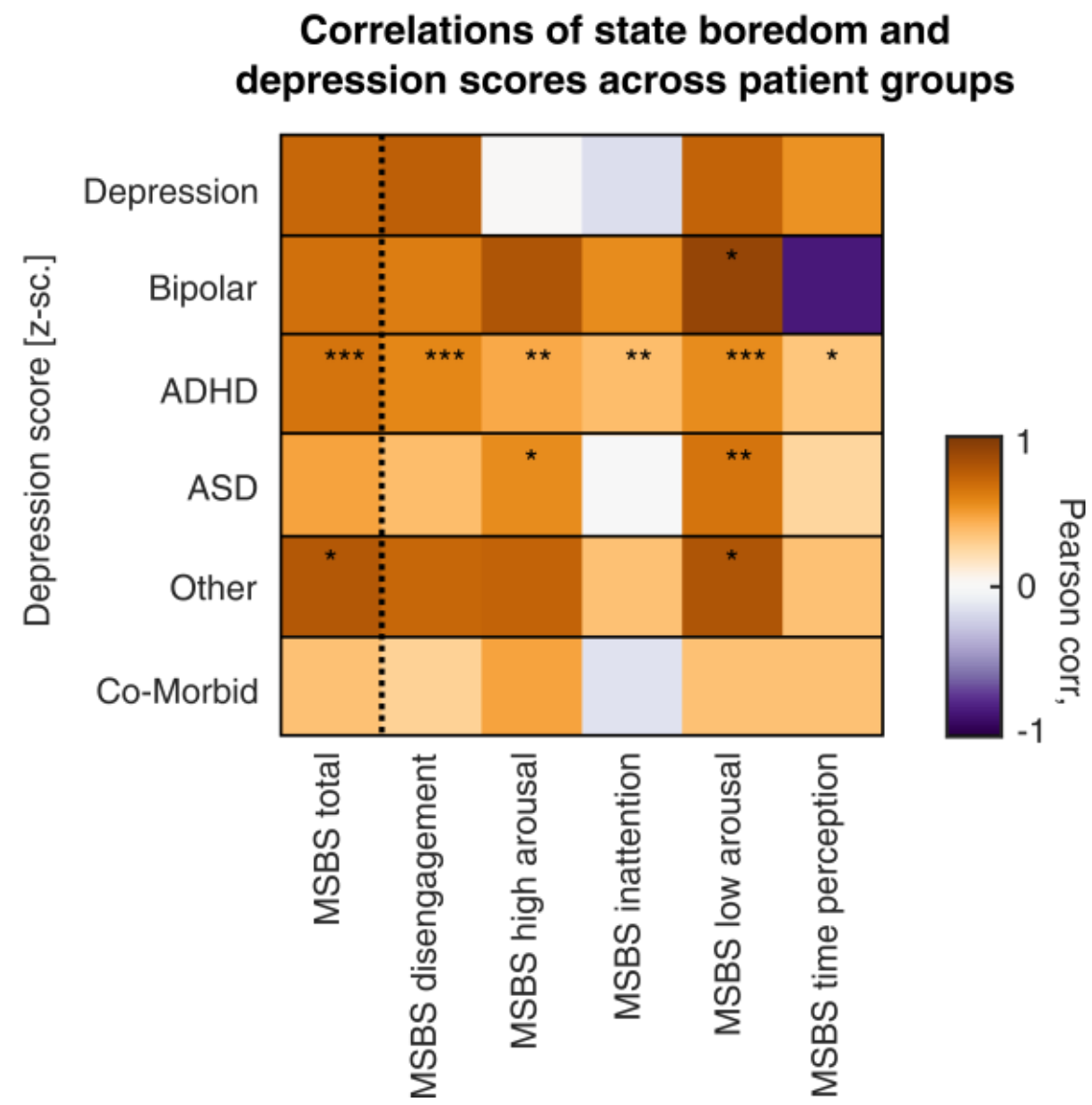

Supplementary Figure 2 – Consistent correlations between state boredom and depressive symptom severity across diagnostic groups: Correlation matrix showing the Pearson correlation coefficients between the state boredom ratings (MSBS total score and sub-scale scores) and the pooled standardized depression scores (see Methods) calculated for each subgroup of patients independently (depression: *n*=7, bipolar disorder: *n*=5, ADHD: *n*=47, ASD: *n*=16, other: *n*=7, co-morbid: *n*=8; numbers refer to all participants that completed the self-reports for this analysis; *: p<0.05, **: p<0.01, ***: p<0.001). The correlation analysis shows a largely congruent correlation pattern across diagnostic groups, supporting the robustness of the positive association between boredom and depression severity.

Supplementary Figure 3

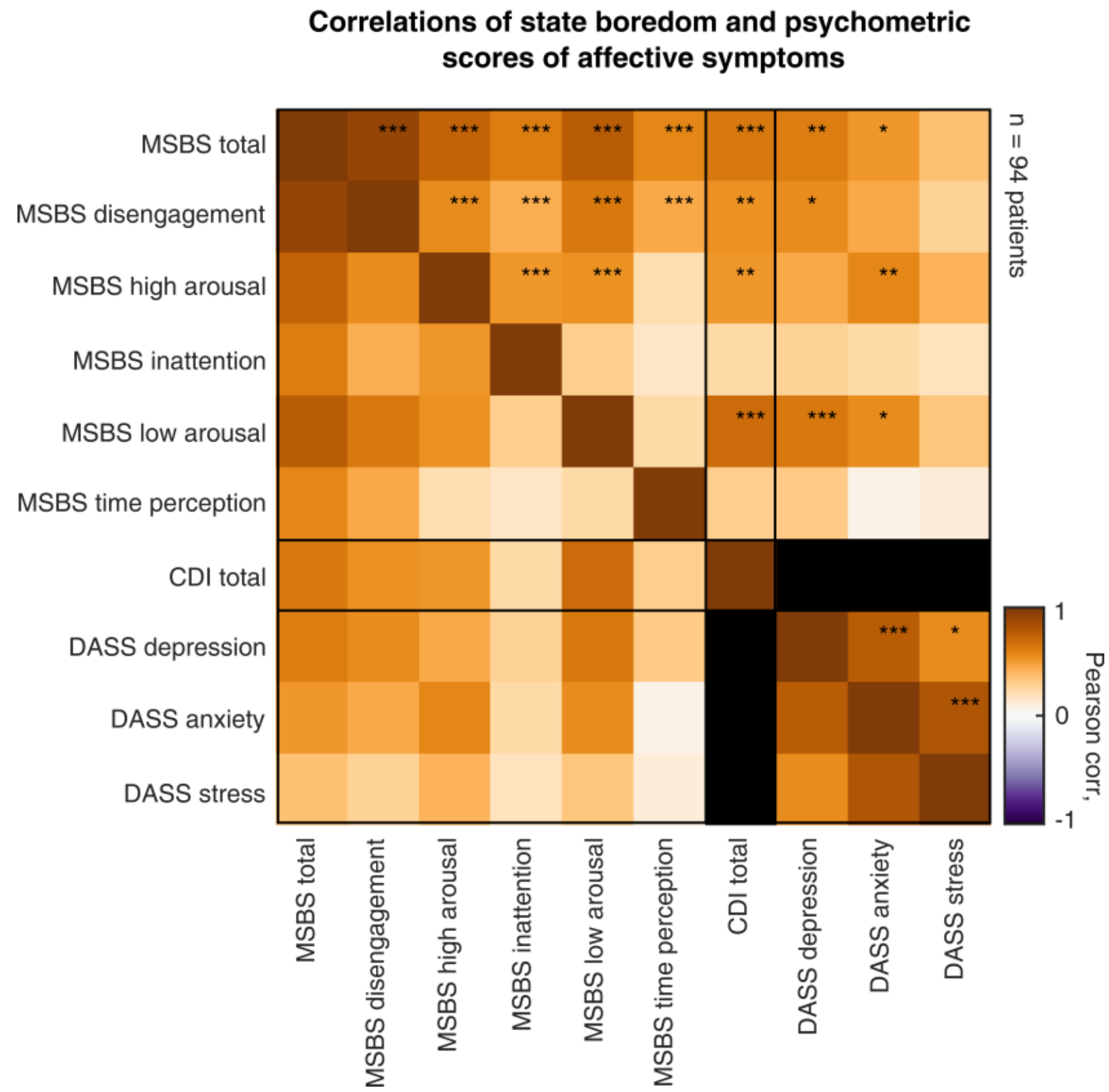

Supplementary Figure 3 – Correlations of state boredom and affective symptoms: Correlation matrix of the state boredom ratings (MSBS total score and sub-scale scores) and psychometric ratings of affective symptoms (CDI and DASS questionnaires; total *n*=94 patients). For each field of the matrix, we computed the correlation with the data of all patients who completed the respective questionnaires (see Methods). Note that CDI and DASS were used in mutually exclusive groups of patients with either <18 years or ≥18 years (see Methods), leaving empty the field in the matrix comparing both metrics (*: *p*<0.05, **: *p*<0.01, ***: *p*<0.001 after Bonferroni-Holm correction for multiple testing). MSBS total scores show a positive correlation with depressive symptom severity measured by CDI or DASS. Among all MSBS subdimensions, altered levels of arousal and disengagement showed the strongest association with depressive symptoms. Abbreviations: DASS: Depression, Anxiety and Stress Scale; CDI: Children's Depression Inventory.

Supplementary Tables

Supplementary Table 1

	**Transdiagnostic patient cohort (total *n* = 94)**
**Major Depressive Disorder (*n* = 8)**	
Age (mean±SD) [years]	22.3 ± 13.8
< 18 years	5 (62.5%)
Female	7 (87.5%)
Male	1 (12.5%)
**Bipolar Disorder (*n* = 6)**	
Age (mean±SD) [years]	27.0 ± 12.5
< 18 years	1 (16.7%)
Female	4 (66.7%)
Male	2 (33.3%)
**ADHD (*n* = 47)**	
Age (mean±SD) [years]	22.9 ± 12.8
< 18 years	25 (53.2%)
Female	18 (38.3%)
Male	29 (61.7%)
**ASD (*n* = 16)**	
Age (mean±SD) [years]	15.6 ± 3.8
< 18 years	11 (68.8%)
Female	4 (25.0%)
Male	12 (75.0%)
**Anxiety* (*n* = 3)**	
Age (mean±SD) [years]	37.3 ± 6.8
< 18 years	0 (0.0%)
Female	1 (33.3%)
Male	2 (66.7%)
**OCD* (*n* = 2)**	
Age (mean±SD) [years]	9.0 ± 1.4
< 18 years	2 (100%)
Female	1 (50.0%)
Male	1 (50.0%)
**Psychosis* (*n* = 3)**	
Age (mean±SD) [years]	25.3 ± 15.3
< 18 years	1 (33.3%)
Female	0 (0.0%)
Male	3 (100.0%)
**Co-morbid (*n* = 9)**	
Age (mean±SD) [years]	21.0 ± 10.8
< 18 years	4 (44.4%)
Female	4 (44.4%)
Male	5 (55.6%)

Supplementary Table 1 – Demographic and psychiatric characteristics of the broad patient sample: Abbreviations: ADHD: Attention deficit hyperactivity disorder; ASD: Autism Spectrum Disorder; OCD: Obsessive Compulsive Disorder; Co-morbid: patients with more than one psychiatric diagnosis. *: Pooled to a group of "other" psychiatric diagnoses in the main analyses.

Supplementary Table 2

Model	n	β interaction	95% CI	t (df)	p	ΔR^2 interaction	Model R^2
**Primary model** (MSBS, depression, MSBS x depression	89	-0.184	[-0.358, -0.010]	-2.10 (85)	0.039	0.033	0.380
**Adjusted model** (+ age, sex, ADHD)	89	-0.197	[-0.361, -0.033]	-2.39 (82)	0.019	0.039	0.476
**CDI subgroup**	49	-0.278	[-0.510, -0.047]	-2.43 (45)	0.020	0.075	0.515
**DASS subgroup**	45	-0.103	[-0.379, 0.172]	-0.76 (41)	0.452	0.011	0.382

Supplementary Table 2 – Sensitivity analyses of the moderation model predicting excessive mind-wandering from state boredom and depressive symptom severity: Results of additional moderation analyses performed to evaluate the robustness of the primary moderation model between mind-wandering, state boredom and depressive symptom severity. The table summarizes the interaction between state boredom (MSBS total scores) and depressive symptom severity (pooled standardized depression scores) in predicting excessive mind-wandering (MEWS scores) for (i) a model adjusted for age, sex, and ADHD diagnosis (ADHD vs. non-ADHD), (ii) the subgroup of participants assessed with the CDI only, and (iii) the subgroup of participants assessed with the DASS Depression scale only. Reported values correspond to the standardized interaction coefficient (β), 95% confidence interval (CI), *t*-statistic, *p*-value, increase in explained variance attributable to the interaction (ΔR^2), and the total model R^2. Abbreviations: CDI: Children's Depression Inventory; DASS: Depression, Anxiety and Stress Scale; MEWS: Mind Excessively Wandering Scale; MSBS: Multidimensional State Boredom Scale.
